# Automated body-lung-air material map segmentation from pre-clinical MRI images for PET attenuation correction in Tera-Tomo 3D PET reconstruction engine of nanoScan PET/MRI system

**DOI:** 10.1186/2197-7364-1-S1-A86

**Published:** 2014-07-29

**Authors:** Peter Bandi, Gabor Jakab, Norbert Zsoter, Domokos Mathe, Gabor Nemeth, Kalman Nagy, Sandor Hobor, Laszlo Papp

**Affiliations:** Department of Software Engineering, Mediso Ltd., Budapest, Hungary; Cromed Research Ltd, Budapest, Hungary; Department of Preclinical Development, Mediso Ltd., Budapest, Hungary

The aim of current work was to automatically derive a body-lung-air three-valued material map from MRI images for pre-clinical PET attenuation correction. Our goal was also to measure the effect of attenuation on corresponding pre-clinical PET images.

12 mouse and 9 rat PET/MRI images were acquired by nanoScan PET/MRI (Mediso). Each MRI image was filtered with edge-preserving Non-Local Means filter.

Two segmentation stages were determined: the first stage separated the body and the air. The gravitational center of each disjunctive region determined by histogram-derived value ranges of the body and the air were inputs of two independent fuzzy-based affinity map generation steps. The fuzzy maps were merged with simple maximum into an initial binary body-air material map. In the second stage the gravitational center of disjunctive air regions laid inside the body mask were the seed points of the third fuzzy affinity map generation. Three fuzzy maps were merged with maximum operation into a three-valued mask.

The three-valued material map was given as input for the Tera-Tomo 3D PET reconstruction engine to serve as the base for attenuation correction.

Mouse and rat material map segmentation result were validated by two experienced pre-clinical researchers who manually drawn the ideal mask boundaries. The ratio of voxel misclassification was measured as 2.23% (mouse) and 1.87% (rat). Quantitative ROI measurements shown 1.3 - 2.7% AC and NAC PET differences.

An automated and robust segmentation method was designed to provide a material map for pre-clinical PET attenuation correction. Although current work operated with PET/MRI images, the same method can be applied for nanoScan SPECT/MRI acquisitions. Results show that on the level of real quantitative measurements, attenuation correction of PET makes sense. As the next step of our future work we will investigate the effects of AC on pre-clinical nanoScan SPECT/MRI images.

